# Out-of-pocket pharmaceutical expenditure and its determinants among Iranian households with elderly members: a double-hurdle model

**DOI:** 10.1186/s12962-024-00521-8

**Published:** 2024-02-19

**Authors:** Mehran Faraji, Tahereh Sharifi, Saeed Mohammad-pour, Javad Javan-Noughabi, Ali Aboutorabi, Shaghayegh yousefi, Mihajlo Jakovljevic

**Affiliations:** 1 Department of Health Economics, School of Management and Medical Information, Iran University of Medical Sciences, Tehran, Iran; 2 Social Determinants of Health Research Center, Semnan University of Medical Sciences, Semnan, Iran; 3 Department of Health Economics and Management Sciences, School of Health, Mashhad University of Medical Sciences, Mashhad, Iran; 4 Social Determinants of Health Research Center, Mashhad University of Medical Sciences, Mashhad, Iran; 5 Kenneth Levene Graduate School of Business, Faculty of Graduate Studies and Research, University of Regina, Regina, SK Canada; 6 UNESCO-TWAS, Trieste, 34100 Italy; 7 Shaanxi University of Technology, Hanzhong, 723099 China; 8 Department of Global Health Economics and Policy, University of Kragujevac, 34000 Kragujevac, Serbia

**Keywords:** Iran, Aged, Health expenditures, Economics, Pharmaceutical, Socioeconomic factors

## Abstract

**Objectives:**

The population of older adults continues to grow in Iran, with pharmaceutical costs as a leading driver of household health-related costs. The present study was conducted to estimate the out-of-pocket pharmaceutical expenditure and its socioeconomic predictors among households with the elderly in Iran.

**Method:**

This study is a secondary analysis using 2019 national household expenditure and income survey data in Iran. The sample size was 9381 households with at least one member older than 65. The double-hurdle model in STATA 16 was used to examine the association between independent variables and households’ out-of-pocket pharmaceutical expenditures.

**Results:**

The mean out-of-pocket pharmaceutical expenditures for each household with elderly member was $8065 per year. There was a positive association between the (female) gender of the household head, urban residence, employment status, insurance expenditure and a higher level of education of the head of the household with the out-of-pocket pharmaceutical expenditures (P < 0.05). The income of elderly households did not affect these expenditures (P > 0.05).

**Conclusions:**

This study showed that the socioeconomic characteristics of elderly families not only influenced their decision to enter the medicine market, but also the rate of medicine purchase. It is helpful to manage and control the pharmaceutical costs among the elderly.

## Introduction

Recently, population pyramid structure have shifted significantly [1]. The relative number of older people, especially those 80 years and older, has increased steadily; a pattern that has come to stay [2]. The peculiarity of the elderly population blotting in the twenty-first century is not without health, economic and social difficulties [3]. Thereby, world health organization (WHO) coined the twenty-first century as the century of the elderly [4]. Worldwide, in 2002, 629 million people (10%) were 60 years of age or older, which will increase to two billion by 2050 and, for the first time in history, the elderly population will outnumber children under 14 years of age [3].

Low and middle income countries were shown to record the highest elderly population growth rate, despite the highest elderly population being reported in high income countries [5]. The increase in the elderly population in high income countries has occurred over 100 to 200 years, while in low and middle income countries, including Iran, this path has occurred over 30 to 40 years [6]. Considering the present elderly population growth rate in Iran, the elderly will constitute about one-third of the population [7]. An aging population requires more health budgets, because health care consumption by the elderly is higher than that of the average members of society [3, 8]. After inpatient and outpatient care, medicines rank third in health care costs; more than one-sixth of health care spending in the Organization for Economic Co-operation and Development (OECD) countries is related to medicine [9, 10]. In Iran, medicine costs were estimated at $4 billion per year [10]. Due to the progression of the disease, polypharmacy and potentially inappropriate medicines (PIMs), the costs of medicine in the elderly are also more significant [11]. A study in Australia showed that more than 81% of the people aged 65 years or older used 1 or more PIMs and it costs an average of US$ 327.07 per person [12].

On the other hand, in most countries, about 40% of pharmaceutical expenditures are covered by out-of-pocket (OOP) payments [13], which is more than 45% in Iran [10]. OOP payments are the sum of patients’ payments for medical services that are not reimbursed by health insurance companies [14–16].

A study showed that elderly people have more medication costs than younger adults. This study found that the mean of the yearly out of pocket pharmaceutical expenditure (OOPPE) were US$192 ± 4.49 for elderly people and US$121 ± 2.57 for younger adults [17].

OOP payments for health care have many harmful effects and prevent large numbers of people from accessing health services [15, 18]. A panel data study in Eastern Mediterranean countries during 2005 to 2013 has shown that high OOP payments can reduce per capita health resources [19]. This study showed that 1% increase in OOP is accompanied with a $12 decrease in health expenditure per capita [19]. Therefore, OOP is known as one of the main factors imposing catastrophic health expenditures on households [20, 21].

The Iranian Food and Drug Administration, a division of the Iranian Ministry of Health (MOH), is in charge of overseeing and controlling the Iranian pharmaceutical industry, which encompasses biological and herbal products [22].

The results of studies about Iran’s pharmaceutical market have showed that medication costs have raised from $2.28 in 1997 to $34.43 in 2010 [23–25]. Starting from 2019, the Iranian government has implemented a comprehensive health insurance scheme, aiming to provide all Iranians with coverage for essential medical expenses [26]. However, the results of a study showed that the availability of about 53% of anticancer medicines was very low in Iran [27]. Sanctions have caused problems in Iran's pharmaceutical industry, including challenges with financial transactions for importing certain medications and the currency's low value compared to the dollar [23, 25].

It has been stated that pharmaceutical pricing is the most important challenge in recent years. Consequently, Iran's pharmaceutical sector has embraced the reference pricing approach to tackle this challenge [24, 25].

In particular, due to the need for more care, the elderly population consumes a higher share of household income [28], and the elderly household is more likely to be exposed to catastrophic and impoverishment health expenditures [4]. Old age is a critical period of life that requires special attention and extensive support to prevent economic and health challenges [29]. Therefore, according to the special role of medicines in managing the health of the elderly, it is necessary to estimate the OOPPE in the elderly. However, there is little empirical knowledge about the household medicine expenditure [10, 30–33]. Therefore, the purpose of this study was to estimate the OOPPE and its predictors among elderly households in Iran to help reduce these costs by identifying the underlying factors.

## Methods

### Database

This is a cross-sectional, descriptive-analytical study. The data for the current study was drawn from the household income and expenditure survey (HIES) of Iran, 2019. HIES is one of the most important statistical surveys that conducted annually in all provinces of Iran to calculate economic indicators. This survey is regularly performed by Iranian Statistical Center (ISC), a part of the Iran Management and Planning Organization. In this survey, the monthly and annual overall expenditures of families for consumer goods before the survey were gathered [34]. In this study, households with at least one elderly person were selected as the research population. The 9381 households that participated in HIES 2019 had one or more person(s) aged 65 or more. We calculate the OOPPE for all members of households that have at least one elderly member.

We extracted the sociodemographic characteristics of the households (e.g., age, gender, and education of household head), household’s income and expenditures (e.g., food, clothing, transport, communication, healthcare, food, tobacco etc.). The OOPPE includes both prescription fees and over-the-counter (OTC) expenditures. We only focused on pharmaceuticals here, thereby excluding dietary supplements. All information on households’ income and expenditure was reported in Rials (Iranian currency). We used 129,000 Iranian Rials as the exchange rate to US Dollar (USD) as declared by the Iranian central bank.

### Analytical framework

Assuming that OOPPE is influenced by participation (whether to spend OOPPE or not) and for econometric and economic reasons, this study used the double-hurdle (DH) model introduced by Cragg (1971). According to the DH model, the household's decision to spend (yes = 1, no = 0) as the first hurdle, and how much to spend as the second hurdle, separately by two sets of explanatory variables were determined [3]. Indeed, we applied a model where the first equation (Probit model) represents the first hurdle, which examines factors influencing a household's decision to spend OOPPE:

$${w}_{i}={z}_{i}\theta +{\varepsilon }_{i}$$

$$d_{i} = \left\{ {\begin{array}{*{20}c} {1,} & {if\, w_{i} > 0} \\ {0,} & {Otherwise} \\ \end{array} } \right.$$

where ${w}_{i}$ is a latent variable representing the household decision to spend OOPPE, ${z}_{i}$ is the vector of covariates, $\theta$ is a vector of coefficients, and ${\varepsilon }_{i}\sim N(0\text{,}1)$.

The second equation examines the optimal amount of OOPPE. According to Cragg (1971), this outcome model can be either a linear model or an exponential model:

$$h_{i} = X_{i} \beta + v_{i} \quad \left( {{\text{Linear}}} \right)$$

$$h_{i} = {\text{exp}}\left( {X_{i} \beta + v_{i} } \right)\quad\left( {{\text{Exponential}}} \right)$$

where ${h}_{i}$ is the latent variable representing OOPPE, ${X}_{i}$ is the vector of covariates influencing OOPPE amount, $\beta$ is the vector of coefficients, and ${v}_{i}\sim N(0\text{,}{\sigma }^{2})$. If both hurdles are passed, then the model is estimated as:

$$y_{i} = \left\{ {\begin{array}{*{20}c} {X_{i} \beta + v_{i} {,}} & {if\,\min \left( {X_{i} \beta + v_{i} {, }z_{i} \theta + \varepsilon_{i} } \right) > 0} \\ 0, & {Otherwise} \\ \end{array} } \right.$$

$$\left( {\begin{array}{*{20}c} {\varepsilon _{i} } \\ {v_{i} } \\ \end{array} } \right)\sim N\left( {0{\text{,}}\,\omega } \right),\,\omega = \left( {\begin{array}{*{20}c} 1 & 0 \\ 0 & {\sigma ^{2} } \\ \end{array} } \right)$$

where ${y}_{i}$ is the observed-dependent variable: OOPPE share, OOPPE, and OOPPE in sub-components. For each equation, we use the Wald test to determine whether the assumption of independence of error terms between participation and expenditure equations is appropriate [4].

The likelihood function of the DH model with dependent error terms across equations takes the form:

$$L = \mathop \prod \limits_{0} \left[ {1 - \phi (\alpha^{\prime}z,\beta^{\prime}x,\rho )} \right] \times \mathop \prod \limits_{ + } \phi \left[ {{{\left( {a^{\prime}z + \frac{\rho }{\sigma }\left( {y - \beta^{\prime}x} \right)} \right)} \mathord{\left/ {\vphantom {{\left( {a^{\prime}z + \frac{\rho }{\sigma }\left( {y - \beta^{\prime}x} \right)} \right)} {\sqrt {1 - \rho^{2} } ]\frac{1}{\sigma }\varphi \left[ {\frac{{y - \beta^{\prime}x}}{\sigma }} \right]}}} \right. \kern-0pt} {\sqrt {1 - \rho^{2} } ]\frac{1}{\sigma }\varphi \left[ {\frac{{y - \beta^{\prime}x}}{\sigma }} \right]}}} \right]$$

where $\phi$ and $\varphi$ denote distributions and density functions.

The goodness of fit test of this model was undertaken by the likelihood ratio test. The Wald (χ^2^) test of independent errors was significant (P > χ^2^ = 0.002), implying rejection of the null hypothesis of zero correlation between error terms of participation and expenditure equations. We used STATA 12 for data analysis and a P-value less than 0.05 is considered statistically significant.

## Results

Among 132,542 Iranian households surveyed in HIES, 9381 households had at least one person over 65 years in the family. Table 1 shows the characteristics of households and the descriptive statistics of the OOPPE among elderly households.

**Table 1:** Characteristics of households and estimation of the OOPPE (USD) among elderly households

Variable	Frequency	Percent	OOPPE	
			Mean	SD
Household head gender				
Female	2611	27.83	3.979	8.809
Male	6770	72.18	6.072	12.764
Household head age				
18–34	135	1.44	5.990	16.460
35–51	494	5.27	7.082	20.025
52–68	2630	28.03	5.764	11.395
69–85	5423	57	5.312	11.114
86 >	700	7.46	4.619	9.761
Living sector				
Urban	4315	46	6.443	13.356
Rural	5066	54	4.677	10.295
Household head marital status				
Single	99	1.06	5.793	13.561
Divorced	94	1	4.745	7.599
Widowed	2947	31.41	3.752	7.115
Married	6241	66.53	6.317	13.444
Household head educational status				
Illiterate	5542	59.08	4.371	8.228
Primary school	2679	28.08	6.753	15.266
Secondary school	598	6.37	8.569	18.666
College and above	562	5.99	7.222	12.516
Household job status				
Housekeeper	482	5.14	5.878	17.802
Unemployed	73	0.78	6.187	8.843
Employed	8826	94.08	5.463	11.443
Ownership				
Free	490	5.22	3.834	6.838
Tenant	349	3.72	6.995	12.010
Owner	8542	91.06	5.523	12.042

6770 (73%) households’ heads were male and 2611 (27%) households had female heads. The average and standard deviation of OOPPE were higher among male-headed households.

Most heads of households were 69–85 year of age (n = 5423, 57%) and the highest average of OOPPE among different age groups of the head of the household was related to the age group of 35 to 51 years ($7.082 ± $20.025). Out of the total households, 4315 (46%) households were rural dwellers and 5066 (54%) were urban inhabitants. The average OOPPE in the rural population ($4.677 ± $10.295) was lower than the average OOPPE in the urban population ($6.443 ± $13.356).

Also, more than 66 per cent of them were married. In this regard, the average OOPPE among elderly households with married household head ($6.317 ± $13.444) was higher than other groups.

In terms of education, 5542 (59.08%) of the heads of the surveyed households were illiterate. The highest average OOPPE was among household’s head with higher education. More than 94% (8826) of the surveyed households had an employed head. The non-employed households’ heads have the highest average OOPPE ($6.187 ± $8.843). While, the 349 households (3.72%) were tenants, but they had the highest average OOPPE ($6.995 ± $12.010).

Of 9381 households in our study, 2988 reported zero OOPPE. The average of OOPPE for each household with elderly was $8065. The minimum and maximum of the OOPPE were $0.155 and $387,596 respectively.

The results of the DH model were shown in Table 2. In the first hurdle, the socio-economic factors affecting the possible participation of the households with elderly (willingness to consume) in the payment of pharmaceutical expenditure were examined. In the second hurdle, the effect of socio-economic factors and the direction of this effect (positive or negative) on the amount of pharmaceutical expenditure were investigated. Household insurance expenses had a small, but significant effect on OOPPE (B = 0.012, P < 0.001). The gender of the household heads has no significant effect on the likelihood of the family taking part in the OOPPE. Household living in rural areas had a negative and significant effect on the probability of household participation in OOPPE (B = − 0.0924, P < 0.001).

**Table 2:** The results of double hurdle model

Pharmaceutical expenditure	First hurdle						Second hurdle					
Explanatory variables	Coefficient	SE	z	P > z	Confidence interval		coefficient	SE	t	P > t	confidence interval	
Income	− 0.0001	0.0002	− 0.4800	0.6290	− 0.0005	0.0003	0.0019	0.0028	0.6700	0.5040	− 0.0036	0.0073
Insurance costs	0.0003	0.0003	0.9200	0.3580	− 0.0003	0.0008	0.0120	0.0031	3.8400	0.0000	0.0059	0.0181
Gender												
Female	Reference											
Male	− 0.0688	0.0570	− 1.2100	0.2270	− 0.1805	0.0428	− 1.2583	0.7229	− 1.7400	0.0820	− 2.6753	0.1588
Age												
18–34	Reference											
35–51	− 0.1289	0.1368	− 0.9400	0.3460	− 0.3970	0.1392	0.0193	1.5654	0.0100	0.9900	− 3.0492	3.0877
52–68	− 0.1921	0.1307	− 1.4700	0.1420	− 0.4484	0.0641	− 0.8020	1.5003	− 0.5300	0.5930	− 3.7428	2.1389
69–85	− 0.1489	0.1309	− 1.1400	0.2550	− 0.4054	0.1077	− 0.2638	1.5023	− 0.1800	0.8610	− 3.2086	2.6811
85 <	− 0.2182	0.1396	− 1.5600	0.1180	− 0.4919	0.0555	− 0.7944	1.6225	− 0.4900	0.6240	− 3.9748	2.3860
Living sector												
Urban	Reference											
Rural	− 0.0925	0.0290	− 3.1900	0.0010	− 0.1493	− 0.0357	− 1.5903	0.3533	− 4.5000	0.0000	− 2.2829	− 0.8977
Marital status												
Single	Reference											
Divorce	− 0.0416	0.1906	− 0.2200	0.8270	− 0.4153	0.3320	-0.7213	2.3443	− 0.3100	0.7580	− 5.3167	3.8742
Widow	− 0.0823	0.1443	− 0.5700	0.5680	− 0.3653	0.2006	− 1.0094	1.7679	− 0.5700	0.5680	− 4.4750	2.4561
Married	0.1234	0.1407	0.8800	0.3810	− 0.1525	0.3992	2.8840	1.7136	1.6800	0.0920	− 0.4750	6.2431
Educational status												
Illiterate	0.0065	0.0617	0.1000	0.9170	− 0.1145	0.1274	− 1.6910	0.7481	− 2.2600	0.0240	− 3.1574	− 0.2245
Primary School	0.0745	0.0620	1.2000	0.2290	− 0.0470	0.1959	0.4380	0.7464	0.5900	0.5570	− 1.0251	1.9011
Secondary School	0.0735	0.0785	0.9400	0.3490	− 0.0803	0.2274	2.0966	0.9355	2.2400	0.0250	0.2629	3.9304
College and above	Reference											
Job status												
Housekeeper	Reference											
Unemployed	0.2773	0.1710	1.6200	0.1050	− 0.0579	0.6125	− 0.1504	2.0160	− 0.0700	0.9410	− 4.1023	3.8014
Employed	0.1841	0.0605	3.0400	0.0020	0.0655	0.3027	− 0.5175	0.7729	− 0.6700	0.5030	− 2.0326	0.9975
Ownership												
Free	Reference											
Tenant	0.1561	0.0936	1.6700	0.0950	− 0.0273	0.3396	2.3569	1.1409	2.0700	0.0390	0.1205	4.5934
Owner	0.0995	0.0612	1.6300	0.1040	− 0.0204	0.2194	1.4053	0.7775	1.8100	0.0710	− 0.1187	2.9294
Number of children below 5 years	0.0909	0.0764	1.1900	0.2340	− 0.0588	0.2407	− 0.5998	0.9073	− 0.6600	0.5090	− 2.3783	1.1786
Family size												
1–2	Reference											
3–5	− 0.0166	0.0332	− 0.5000	0.6160	− 0.0817	0.0484	− 0.2076	0.4031	− 0.5200	0.6070	− 0.9977	0.5825
6 <	0.0338	0.0679	0.5000	0.6190	− 0.0993	0.1670	− 0.6883	0.8141	− 0.8500	0.3980	− 2.2840	0.9074
Constant	0.3806	0.1941	1.9600	0.0500	0.0002	0.7611	2.1971	2.3315	0.9400	0.3460	− 2.3732	6.7673
LR chi2	92.89						244.71					
Probability	0						0					
Pseudo R^2^	0.0079						0.0043					
Log likelihood	− 5823.6757						− 28,616.061					

The household head occupation had a significant effect on the likelihood of the family taking part in the OOPPE (B = 0.184, P < 0.001), however, it has no significant effect on their OOPPE. Ownership of rented housing had a positive and significant effect on OOPPE (B = 2.356, P ≤ 0.05). The findings of this study showed that the income of elderly households does not affect the probability of household participation in OOPPE and the OOPPE. Male family headship had a negative and non-significant effect on the OOPPE (B = − 1.297, P = 0.07). In addition, households living in rural areas had a negative and significant effect on OOPPE (B = − 1.590, P < 0.001). While, household head illiteracy had a negative and significant effect on the OOPPE (B = − 1.690, P < 0.05; secondary school education significantly affect OOPPE (B = 2.096, P < 0.05). Increasing the family size had a positive and non-significant effect on participation in OOPPE. But it had a positive and non-significant effect on the OOPPE.

## Discussion

Health care interventions are effective in treating diseases and improving the quality of life of patients; however, these interventions can lead to imposing costs on patients [35–37]. In the present study, we reported that Iranian households with an elderly person pay out of pocket an average of $8065 for medicine annually, erstwhile recorded as $92.17 per senior [6]. The aged expend more on medicine [7], similar findings were obtained in the United States [9], Australia [8] and Canada [14]. Given the fact that leading Emerging BRICS markets are the engine of global real GDP growth it is important to notice that their long term health spending patterns follow the comparable pathways [38]. High pharmaceutical expenditure in the elderly can be due to the prevalence of chronic non-communicable diseases (NCD) [39] and the increase in polypharmacy phenomenon [8, 11, 15]. Which is associated with increased demand and thus increase pharmaceutical expenditure in the elderly [17]. Additionally, seniors were usually consulted by specialist physicians which have cost more [7, 18]. Therefore, considering the doubling of the elderly population in Iran during 2015 to 2030, an effective referral system along with the implementation of NCD prevention and control strategies in the country, can help reduce pharmaceutical expenditure in the elderly [7].

On the other hand, the pharmaceutical expenditure cannot be a good measure alone because the pharmaceutical expenditure does not reflect the “unmet needs”, that is some medicines prescribed for some households are not easily available and therefore not purchased [40]; hence, we use double hurdle model in this study to tackle this shortcoming. In Australia in 2014–2015, about 30% of households in the lowest income households had no OOP payments for medicine, while in the richest income group this figure was 11%, for some vulnerable groups, automatic exemptions and OOP payments have been set [41]. In several western countries, medical expenses for seniors have been abolished or reduced for individuals, depending on their age or income [17]. Studies from the USA and South Korea reported high OOP payments that low-pay individuals spend more than they procure on health care [17]. Bearing in mind the Ando and Modigliani life cycle theory, which proves that a person's income stream is low at the beginning and end of life and more in the middle of life [42]. The country's policy efforts are expected to come up with modalities to save the middle-aged income by injecting it as increased welfare subsidies, such as community-based health insurance in old age [43].

Our study shows that rural dwelling had a negative and significant effect on the willingness to pay and the amount of household medical expenses; Dizal et al.’s study reported similar findings [7]. Rural dwellers in Iran have full insurance coverage and they frequently patronize government services. In addition, traditional medicine (complementary: alternative medicine), still has a special place in Iranian culture, especially in rural areas. According to studies, villagers (especially in chronic diseases) are the most users of complementary medicine [44].

We found that the marital status of the head of the household had no significant effect on the average medication expenditure among elderly households. This result contrast the findings in China and Austria [8–10]. Accordingly, higher education of the caregiver significantly increases the household medicine expenses. Previous studies have shown that educated people spend more on pharmaceuticals [7, 16]; likely due to their knowledge and awareness of better choices [45]. Further, household head employment status had a significant effect on the tendency of households with the elderly to consume, but no effect on household medical expenditures, while the expenditures of the unemployed were found to be higher.

Homeownership has a significantly higher health spending effect on a household, however, Savojipour and colleagues reported no different health spending between tenants and homeowners [46]. Herein, tenant households had higher OOP payments for medicine than other groups probably due to their inability to afford the insurance prepayment system.

Here, we found male household heads spent significantly lower on health care compared to females. A study reported that female household heads in Iran are poorer than their male counterparts [47]. Men have a stronger economic security margin due to the possibility of multiple employment and higher income, while the women were unable to pay for health insurance, they pay OOP for higher pharmaceutical costs. In Ethiopia [48] and Austria [2] female-headed households spend more on OOP payments, while the opposite was the case in Spain [30].

Household insurance expenses had a small but significant effect on household medicine expenses. Of note, basic health insurance could not prevent Iranian households from incurring devastating health costs [49]. While in Spain and China, having health insurance was a predictor of reduced pharmaceutical expenditure for inpatients and outpatients [16, 30]. Whilst, people with insurance are expected to spend less on medicine, health insurance in Iran does not significantly reduce the burden. Weaknesses in the insurance support system can lead to OCT medicine supply and self-medication resulting in physical complications [50]. In general, treatment goals selection, patient education, generic medicines utilization and female heads empowerment are among the basic measures to reduce pharmaceutical expenditure [51].

We found that the income of elderly households does not affect the willingness to spend on pharmaceutical expenditure and the OOPPE [52]. In Iran pharmaceutical expenditure is unaffected by the increase in household income [40]. Similarly, pharmaceutical expenditure is unaltered by the household income status in Catalonia [30]. In contrast, in China, higher-income groups tend to spend less on medicines and pay less OOP [16]. One of our limitations was that we don’t investigate some variables such as number and type of diseases of the elderly due to lack of access to these variables. Although the decision to use drugs may be influenced by household characteristics, it is ultimately an individual decision and primarily depends on individual characteristics. Therefore, it is necessary to be careful in generalizing the results of the household level to the individual level.

## Conclusion

The findings of this study showed that OOPPEs are significant among household with elderly members. Additionally, the findings of the double hurdle model indicate that socio-economic factors significantly influenced the likelihood of households with elderly members participating in the payment of pharmaceutical expenses, as well as the amount of pharmaceutical expenditure.

Identifying these factors is very important for policy makers and health system planners and can be useful and effective in determining the best policies for controlling and managing health expenditures. Therefore, according to the growing trend of the elderly population in Iran, it is necessary to formulate policies and measures to face the increasing phenomenon of aging and improve the socio-economic situation in order to reduce pharmaceutical expenses and inequality in the health sector.

This can be achieved through job creation, especially in female-headed households, investing in self-care education, NCDs prevention and control strategies for future generations, developing incentive policies for prescribing generic medicines, full implementation of family physicians and referral system with modification of supply behavior.

## Data Availability

The datasets used and/or analyzed during the current study are available from the corresponding authors on reasonable request.

## Abbreviations

WHO: World health organization
OECD: Organization for Economic Co-operation and Development
PIMs: Potentially inappropriate medicines
OOP: Out-of-pocket
OOPPE: Out of pocket pharmaceutical expenditure
HIES: Household income and expenditure survey
ISC: Iranian statistical center
OTC: Over-the-counter
DH: Double-hurdle
